# Pustular Rosacea in an 8‐Year‐Old Patient: A Rare Presentation of Pediatric Rosacea

**DOI:** 10.1155/crdm/6001322

**Published:** 2026-04-27

**Authors:** Razan Sharaf, Tasneem Assaf, Usra I. Ghanem, Hadeel Abed Alhameed, Rabee Adwan

**Affiliations:** ^1^ Faculty of Medicine, Al-Quds University, Jerusalem, State of Palestine, alquds.edu; ^2^ Pediatric Department, Al-Makassed Hospital, Jerusalem, State of Palestine; ^3^ Infectious Department, Al-Makassed Hospital, Jerusalem, State of Palestine; ^4^ Infectious Disease Unit, Makassed Hospital, Al-Quds University, Jerusalem, State of Palestine, alquds.edu

**Keywords:** inflammatory diseases, pustular rosacea, rash

## Abstract

Pustular rosacea is a rare chronic inflammatory disease whose prevalence increases with age. We report a case of an 8‐year‐old patient who presented with a 3‐year history of recurrent papulopustular facial eruptions associated with severe burning sensation, primarily affecting the cheeks, nose, and chin, exacerbated by hot weather and swimming. His condition was misdiagnosed as psoriasis, eczema, food allergy, or acne with limited benefit from treatment other than temporary relief from glucocorticoids. Skin biopsy showed hyperkeratosis and vascular ectasia with neutrophilic microabscesses consistent with papulopustular rosacea. He was successfully managed with lifestyle modifications and medications including azelaic acid, metronidazole, and ambroxol hydrochloride resulting in significant improvement. Diagnosing rosacea can be challenging due to its overlap with other conditions. Early diagnosis and treatment are crucial to prevent complications like scarring, persistent erythema, and psychological distress while ensuring a better quality of life and sustained remission.

## 1. Introduction

Pustular rosacea is a rare dermatologic presentation of a chronic inflammatory skin disease that predominantly affects adults, with incidence increasing significantly with age [1].

Although rosacea as a whole is most common in adults—particularly those aged 30–50 years—its occurrence in children is uncommon, making pediatric cases more difficult to recognize and diagnose [2]. Diagnosis is primarily clinical and relies on characteristic symptom patterns [2]. Phymatous, papulopustular, ocular rosacea, and erythematotelangiectatic are the four kinds that have historically been recognized [2]. Since rosacea is a chronic condition with a cycle of flare‐ups and remissions, lifestyle changes and avoiding triggers are the mainstays of treatment [1]. Depending on the clinical characteristics and severity of the condition, pharmacological therapy—topical alone or in conjunction with systemic pharmacological treatment—is used [3].

The complicated pathophysiology of rosacea includes skin microbiome imbalance, inherent and adaptive immune system dysregulation, nerve and blood vessel dysfunction, and the interaction of hereditary and environmental variables [1]. Children can also get rosacea, but it mostly affects adults, especially middle‐aged women [4].

Rosacea is known to cause local side effects, such as lupoid granulomas, papular and aseptic pustular lesions, telangiectasias, seboglandular hyperplasia, and persistent facial problems. Early diagnosis and treatment are therefore important to minimize these outcomes [5].

In this report, we describe the successful treatment of inflammatory rosacea in an 8‐year‐old child using a combination of topical and systemic therapy. Presenting this case aims to raise awareness among physicians and encourage considering pustular rosacea in the differential diagnosis of pediatric pustular rashes.

## 2. Case Presentation

An 8‐year‐old male child presented to our clinic with a 3‐year history of recurrent facial eruption, localized to the central face, particularly on the cheeks, nose, and chin. The lesion started as a gradual, small red papule limited to his cheeks that evolved to become multiple inflamed pustules and spread to other facial areas, but without body involvement or telangiectasia. The papulopustular rash was associated with a severe burning sensation. At the same time, the patient complained of coughing and mucus secretion.

**FIGURE 1:**
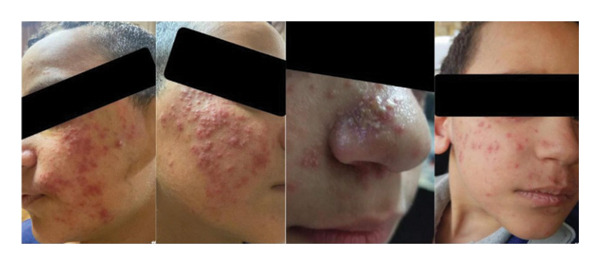
Pustular pattern progression during treatment course.

Over the past 3 years, he has been experiencing episodes of severe flare‐ups that sometimes may be accompanied by fever followed by periods of remission, but his symptoms never completely resolved. His condition was exacerbated when he was exposed to the hot weather and swimming. Only topical glucocorticoids helped in decreasing his symptoms. The child is otherwise healthy, with no history of fatigue, weight loss, or other systemic symptoms. He had no known drug–food allergies, and both past medical and surgical history were free. The family denies any significant history of trauma, stress, insect bites, medication, or chemical exposure that could have triggered the eruption. His father was diagnosed with rosacea at the same age, and he has adhered to his medication and lifestyle modifications since that time. On examination, the child appeared well and active with normal body temperature. He had erythematosus, inflamed papules and pustules located primarily on the cheeks, nose, and chin (Figure 1) without involving other body parts. There was no evidence of scarring or significant comedones. The lesions were tender to touch but did not show signs of crusting or ulceration. Otherwise, the child is healthy, and systemic examinations were normal, with no abnormalities. He was seen by 12 different dermatologists without receiving a clear diagnosis, so the family decided to proceed with a biopsy despite his young age. The patient previously had many misdiagnoses, including psoriasis, eczema, food allergies, and acne vulgaris. Numerous treatments were tried including topical benzoyl peroxide and isotretinoin without any benefit. Only topical cortisone had a short‐term impact.

**FIGURE 2:**
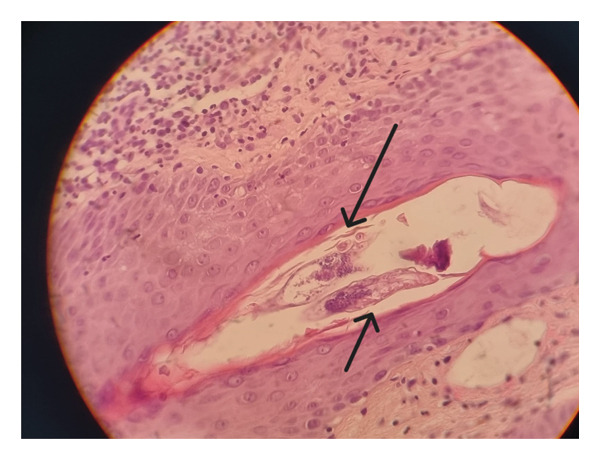
Skin punch biopsy showing *Demodex* mites.

Two skin punch biopsies were taken; each measured 0.2 × 0.2 × 0.3 cm. Both sections showed similar features but are more pronounced in one of them. There was hyperkeratosis, hypergranulosis, and acanthosis with minimal spongiosis and exocytosis. *Demodex* mites were seen in all hair follicles present (Figure 2). The dermis shows vascular ectasia with multiple collections of neutrophils (microabscesses) surrounded by moderately dense perivascular lymphohistiocytic cellular infiltration. No true granulomas were evident. Moreover, the periodic acid–Schiff (PAS) stain for fungi and Ziehl–Neelsen (ZN) stain for acid‐fast bacilli were both negative.

Finally, the patient was diagnosed with papulopustular rosacea managed by lifestyle modification such as avoiding sun exposure and the following medications: azelaic acid 15% for redness, topical metronidazole 0.75% twice daily for rosacea, topical ivermectin 10 mg/g for mites, and ambroxol hydrochloride 30 mg three times daily for the cough. His symptoms gradually improved. The lesions were resolved after 1 month. At 3‐year follow‐up, there are no lesions and the patient is doing well.

## 3. Discussion

Rosacea is a common inflammatory disease that affects the nose, cheeks, chin, and forehead.

It presents with erythema, flushing, papules or pustules, and telangiectasia. In pustular rosacea, it commonly presents as flushing of the face and then progresses to papules and pustules [1]. There are four known subtypes of rosacea: papulopustular, phymatous, erythematotelangiectatic, and ocular rosacea. These subtypes are based on the clinical signs and symptoms [6]. Phymatous rosacea is not seen in pediatrics, but an additional subtype is seen in infants and children which is idiopathic facial aseptic granuloma [2]. The etiology of rosacea is not well established. It is known that genetics, immune reactions, environmental factors, neurovascular dysregulation, and ultraviolet light can contribute to the disease [7].

Rosacea affects an estimated 5% of the global population and occurs most frequently in adults aged 30–50. Pediatric rosacea, including the pustular subtype, is rare, and its true incidence may be underestimated due to frequent misdiagnosis and the absence of a diagnostic gold standard [8]. Papulopustular rosacea may resemble acne, earning the nickname “adult acne,” but the absence of comedones helps differentiate the two conditions [9].

Our patient was misdiagnosed with many conditions including adult acne, contact dermatitis, and eczema. The similarity of the lesions made pustular rosacea challenging to diagnose.

According to the latest guidelines established by the National Rosacea Society Expert Committee, the diagnosis requires the presence of one of the following features: (a) characteristic fixed centrofacial erythema that may intensify periodically or phymatous changes (b) or at least two of the following major criteria: flushing, pustules and papules, telangiectasia, and/or ocular manifestations [10]. Laboratory tests and cultures play no role in diagnosing rosacea, and a skin biopsy is limited. Despite that, some studies conducted biopsies in patients with rosacea, which concluded little significance in the diagnosis and treatment [9].

The current treatment of rosacea is designed to improve the quality of life of the patient as it is chronic and relapses. Identification of the subtype and the triggers can enhance individualized treatment. For pustular rosacea, topical solutions have been proven to be useful. In a 2015 Cochrane review, topical metronidazole, azelaic acid, and ivermectin were all effective in papulopustular rosacea, especially ivermectin [11, 12].

For systemic therapies, oral antibiotics such as metronidazole are the treatments of choice in pustular rosacea. However, in persistent cases of pustular and early phymatous rosacea, low‐dose isotretinoin (0.3 mg/kg daily) is effective. Yet, relapse after discontinuation of isotretinoin is common [13]. Nonetheless, our patient was treated with azelaic acid, metronidazole, ambroxol hydrochloride, and ivermectin and has improved significantly.

The differential diagnosis of rosacea is acne, seborrheic dermatitis, keratosis pilaris rubra, flushing, acute cutaneous lupus erythematosus, and drug‐induced acneiform eruption.

It is important to distinguish between these conditions, establish suitable management, improve the quality of life of the patient, and sustain remission [9].

Acne is characterized by the presence of papules, pustules, and comedones. The absence of comedones in our patient helped us to exclude acne. As for seborrheic dermatitis, it features erythema and greasy scaling on the face and scalp, in addition to the typical distribution in the nasolabial folds and hair‐bearing areas of the face. The presence of the pustules, not erythema, and the uncharacteristic distribution of seborrheic dermatitis made it unlikely to be in our patient.

Keratosis pilaris rubra involves tiny follicular papules at the lateral cheeks and erythematosus patches on the neck. It is not the case here as there is no involvement of the neck. Furthermore, flushing in cases of rosacea involves the face only; flushing of other areas suggests other etiologies. In acute cutaneous lupus erythematosus, the rash appears familiar to rosacea except for sparing the nasolabial folds and lacking pustules. Finally, drug‐induced acneiform eruption is usually related to drug intake and is sudden in onset and involves papulopustules that are in the same stage as well as trunk involvement [9].

The prognosis of rosacea is good overall. Despite that, if left untreated, it can lead to scarring and persistent erythema, which may lead the patients to develop anxiety and depression. Recent studies have shown the involvement of cardiovascular, gastrointestinal, and endocrine comorbidities in adults [14]. Although pediatric rosacea is rare, the most common subtype reported is papulopustular rosacea, and most cases involved a positive family history [3].

Finally, this article highlights that pustular rosacea is a chronic dermatological condition that can occur in children presenting as papules and pustules in the face; it is important to rule out other papulopustular disorders as well. We recommend keeping pustular rosacea in mind when cases present with pustules.

## Funding

No funding was received for this manuscript.

## Consent

Written consent for publication of medical information and images was obtained from the patient’s guardian.

## Conflicts of Interest

The authors declare no conflicts of interest.

## Data Availability

The data that support the findings of this study are available from the corresponding author upon reasonable request.
